# Targeted Therapies for Pediatric AML: Gaps and Perspective

**DOI:** 10.3389/fped.2019.00463

**Published:** 2019-11-15

**Authors:** Annalisa Lonetti, Andrea Pession, Riccardo Masetti

**Affiliations:** ^1^“Giorgio Prodi” Interdepartmental Cancer Research Centre, University of Bologna, Bologna, Italy; ^2^Pediatric Hematology-Oncology Unit, Department of Medical and Surgical Sciences DIMEC, University of Bologna, Bologna, Italy

**Keywords:** Pediatric AML, targeted therapy, FLT-3 inhibitors, Hedgehog pathway inhibitors, DOT1L inhibitors

## Abstract

Acute myeloid leukemia (AML) is a hematopoietic disorder characterized by numerous cytogenetic and molecular aberrations that accounts for ~25% of childhood leukemia diagnoses. The outcome of children with AML has increased remarkably over the past 30 years, with current survival rates up to 70%, mainly due to intensification of standard chemotherapy and improvements in risk classification, supportive care, and minimal residual disease monitoring. However, childhood AML prognosis remains unfavorable and relapse rates are still around 30%. Therefore, novel therapeutic approaches are needed to increase the cure rate. In AML, the presence of gene mutations and rearrangements prompted the identification of effective targeted molecular strategies, including kinase inhibitors, cell pathway inhibitors, and epigenetic modulators. This review will discuss several new drugs that recently received US Food and Drug Administration approval for AML treatment and promising strategies to treat childhood AML, including FLT3 inhibitors, epigenetic modulators, and Hedgehog pathway inhibitors.

## Introduction

Acute myeloid leukemia (AML) is a hematological malignancy characterized by the clonal expansion of myeloid precursors, which acquire genetic abnormalities in cellular components involved in self-renewal, proliferation, and differentiation. Pediatric AML accounts for ~25% of pediatric leukemias and although relative low frequent, it represents a clinical challenge, due to its poor prognosis. Over the last 20 years, considerable improvements in overall survival (OS) were achieved, mainly due to intensified treatment strategy, enhancements in supportive care and progresses in risk-adapted patient stratification. Despite that, OS does not exceed 70%, and relapse rates range between 25 and 35% (1), highlighting an urgent need for novel treatments. AML has an age-related profile, with regard to either incidence rate or genetic features. Indeed, the incidence of pediatric AML peaks in infants (children aged 0–1 years), and it is rare in children up to 18 years and further increases in persons between 18 and 60 years of age (2). In addition, genetic abnormalities that occur in infants distinguish a peculiar subgroup of patients (3).

In recent years, with advances in technology, there have been tremendous progresses in defining the molecular landscape of AML, and novel AML entities were included in the most recent World Health Organization (WHO) classifications (4), including AML with *NPM1* or *CEBPA* mutations. However, the occurrence of cytogenetic abnormalities as well as genetic mutations identifying specific WHO entities (e.g., *NPM1, FLT3, CEBPA* mutations) is lower in pediatric than in adult AML, and a high percentage of pediatric patients (>40%) fall in the “AML not otherwise specified” (AML-NOS) category, thus limiting the applicability of WHO classification in children with AML (5). Furthermore, thanks to the recent sequencing approaches, major insights into pediatric AML genetic alterations, distinct from those of adult AML, were achieved. Importantly, these findings greatly affected patient risk stratification and provided new therapeutic targets (6). In this regard, in 2018, Bolouri et al. published the results of the TARGET project, reporting a comprehensive analysis of the molecular aberrations occurring in a large cohort of pediatric AML (7). The main features of pediatric AML emerged from this study were a low overall mutation rate, likewise adult AML, but a landscape of somatic aberrations different from that observed in adult patients, and including structural changes, aberrant DNA methylation, and novel pediatric-specific mutations in genes characteristically mutated in AML. More specifically, the most common mutated genes in pediatric AML included *RAS, KIT*, and *FLT3*, and novel pediatric-specific *FLT3* mutations were identified. Conversely, *DNMT3A, IDH1*, and *IDH2* gene mutations were nearly absent in pediatric AML. Novel focal deletions were identified in *IL9R, MBNL1*, and *ZEB2* genes, and further deletions affected *ELF1* expression. A variety of fusion genes were detected, many of which were primarily or exclusively associated to pediatric AML, for instance, *CBFA2T3-GLIS2* and *NUP98-NSD1*. Also, multiple epigenetic regulators, particularly *KMT2A* and *WT1*, were affected by both structural and mutational anomalies. Interestingly, the associated epigenetic changes induced transcriptional silencing of activating ligands for natural killer (NK) cells or genes that converge on Wnt–β-catenin signaling, both representing potential therapeutic targets. The most remarkable information emerged from the TARGET study were the age-related distribution of genomic anomalies and the interactions among mutations that have clinical consequences, thus demonstrating the importance to improve the identification of genomic alterations to better stratify pediatric AML patients as well as to develop novel targeted therapies. Indeed, at present, all the information regarding the molecular landscape of AML marginally resulted in novel therapeutic strategies, and in the last decades, with a few exceptions, there was a general stagnation in standard chemotherapeutic approaches. Fortunately, this scenario is gradually changing (8). In this review, we provided an overview of several therapeutic approaches to target specific genetic lesion of pediatric AML, with special attention on drugs that recently received US Food and Drug Administration (FDA) approval for AML treatment together with promising strategies to treat definite subgroup of pediatric AML. A summary of selected inhibitors discussed in the present review and currently investigated in pediatric AML is provided in Table 1.

**Table 1:** Targeted inhibitors in clinical trials for pediatric AML.

**Target**	**Drug**	**Intervention**	**Condition**	**Phase**	**Age group**	**Clinical trial identifier**	**Status**
FLT3	Sorafenib	Sorafenib in combination with chemotherapy	*De novo* AML	III	Up to 29 years (child, adult)	NCT01371981	Completed
		Sorafenib in combination with idarubicin and Ara-C	Diagnosis AML and high-risk MDS	I–II	15–60 years (child, adult)	NCT00542971	Completed
		BTK inhibitor with chemotherapy with/without Sorafenib	Refractory/relapsed FLT3 mutant AML	II–III	14–60 years (child, adult)	NCT03642236	Recruiting
		Sorafenib in combination with cytarabine and clofarabine	Refractory/relapsed hematologic malignancies	I	Up to 31 years (Child, Adult)	NCT00908167	Completed
		Palbociclib and Sorafenib, Decitabine, or Dexamethasone	Recurrent or refractory leukemia	I	15 years and older (child, adult)	NCT03132454	Recruiting
		Sorafenib	Refractory/relapsed solid tumors or leukemia	I–II	2–21 years (child, adult)	NCT01445080	Completed
	Lestaurtinib	Lestaurtinib in combination with cytarabine and idarubicin	Refractory/relapsed FLT3 mutant AML	I–II	1–30 years (child, adult)	NCT00469859	Completed
	Midostaurin	Midostaurin in combination with standard chemotherapy	*De novo* FLT3 mutant AML	II	3 months to 17 years (child)	NCT03591510	Recruiting
		Midostaurin	Relapsed/refractory acute leukemias (MLL-rearranged ALL ad FLT3 mutated AML)	I–II	3 months to 18 years (child, adult)	NCT00866281	Completed
	Quizartinib	Quizartinib in combination with re-induction chemotherapy and as a single-agent maintenance	Refractory/relapsed FLT3 mutant AML	I–II	1 month to 21 years (Child, Adult)	NCT03793478	Recruiting
	Crenolanib	Crenolanib in combination with Sorafenib	Refractory/relapsed FLT3 mutant AML	I	1 year to 39 years (Child, Adult)	NCT02270788	Completed
	Gilteritinib	Gilteritinib in sequential combination with chemotherapy	Refractory/relapsed FLT3 mutant AML	I–II	6 months to <18 years of age (and young adults)	2215-CL-0603	Planned
		Gilteritinib in sequential combination with chemotherapy	Newly diagnosed FLT3 mutant AML	II	6 months to <18 years of age (and young adults)	2215-CL-0604	Planned
DOT1L	Pinometostat	Pinometostat	Relapsed/refractory leukemias with *MLL* rearrangements	I	3 months to 18 years (child, adult)	NCT02141828	Completed
		Pinometostat with standard chemotherapy	Newly diagnosed AML with *MLL* Rearrangement	I–II	14 years and older (child, adult)	NCT03724084	Recruiting
KIT	Dasatinib	Dasatinib in consolidation therapy in CBF-AML	*De novo* AML	N.A.	6 months to 16 years (child)	NCT03173612	Recruiting
		Dasatinib in combination with chemotherapy	Relapsed t(8;21) AML With *KIT*^D816^ mutation	I	Child, adult, older adult	NCT03560908	Recruiting
CD33	Lintuzumab	Lintuzumab	Relapsed/refractory AML	I	16 years and older (child, adult, older adult)	NCT00002890	Completed
		Actinium-225 labeled to lintuzumab	Relapsed/refractory AML	I	Child, adult, older adult	NCT00672165	Completed

*BTK, Bruton's tyrosine kinase; MLL, mixed-lineage leukemia; FLT3, fms related tyrosine kinase 3; DOT1L, disrupter of telomeric silencing 1-like histone methyltransferase; AML, acute myeloid leukemia; ALL, acute lymphoblastic leukemia*.

## Targeting Gene Mutations: Focus on *FLT3* and *KIT*

AML development is a multistep process that requires the cooperation of at least two genetic abnormalities, classified as type I (that confer a proliferation advantage on hematopoietic cells) and type II alterations (that impair hematopoietic differentiation) (9). These anomalies include both karyotypic alterations and gene mutations, with the latter frequently occurring in cytogenetically normal AML. Although AML is a cancer with a very low rate of somatic alterations, because of the constant identification of novel recurrent gene mutations, nowadays more than 90% of pediatric AML are identified to have at least one genomic alteration (10), among which those affecting *FLT3* and *KIT* genes are very common in children, with more than 20% and 10% frequency, respectively, according to the TARGET study (7).

FLT3 is a transmembrane type III receptor tyrosine kinase that is activated by the specific FLT3 ligand and, subsequently, regulates hematopoiesis through phosphorylation of downstream targets, including STAT5, and activation of critical oncogenic pathways such as Ras/Raf/MAPK and PI3K/Akt/mTOR (11). Activating mutations of FLT3 include both internal tandem duplication (FLT3-ITD) and point mutations of the activation loop domain (FLT3-TKD), with a prevalence of ~15 and 7%, respectively, in pediatric AML (12). Ligand-independent FLT3 activation leads to a decreased maturation and an increased proliferation of myeloid progenitors. Importantly, FLT3 mutations are prognostically relevant in pediatric AML, and the presence of ITD particularly with an high allelic ratio (AR) of ≥0.5 have a prognostic impact and are significant predictive factors for an adverse outcome (12–14). Therefore, FLT3 mutated pediatric AML patients are considered high risk and, nowadays, they are offered allogenic hematopoietic stem cell transplantation (HSCT) in first complete remission (15). The use of HSCT can override the negative prognostic impact of FLT3 mutations, as demonstrated by similar probability of 8-year event free survival (EFS) in both FLT3-ITD and wild-type subgroups (15). However, there are potentially severe side effects correlated to this procedure, and there is still a consistent proportion of patients not eligible for HSCT, thus supporting the relevance to improve current treatments for FLT3 mutated patients. In addition, FLT3 mutations, even if not detectable at diagnosis, can subsequently appear at relapse because of clonal selection, and may further affect prognosis (16). Given the high number of both adult and pediatric AML patients harboring FLT3 mutations (7, 17) and their poor outcome, many efforts have been made to develop FLT3 targeted inhibitors, and a variety of compounds have entered clinical trials for both adult and pediatric patients (Table 1). The first generation of FLT3 inhibitors, which entered clinical trials since the early 2000s, were not FLT3 specific but targeted multiple kinases. In pediatric AML, the most extensively studied first-generation FLT3 inhibitor is Sorafenib, which was investigated as single agent or in combination with other drugs in several formal clinical trials enrolling both *de novo* or refractory/relapsed AML. In pediatric AML, the MTD of Sorafenib was defined as 150 mg/m^2^ (18, 19). Sorafenib showed a significant antileukemic activity in relapsed or refractory pediatric AML, inducing a reduction by more than 50% of bone marrow blasts and, in combination with clofarabine and cytarabine, it achieved a complete remission in 8 out 12 patients, including both wild type and mutated FLT3 (18). As a single agent, the activity of Sorafenib was observed in 2/8 pediatric refractory AML, both with FLT3-ITD (19). Importantly, in these trials, remission achievement allowed to proceed with allergenic HSCT. These findings resulted in further investigation of Sorafenib in newly diagnosed or FLT3 mutant AML (NCT01371981, NCT03642236). Other first-generation FLT3 inhibitors evaluated for pediatric AML treatment include Sunitinib (20), Lestaurtinib (NCT00469859), and Midostaurin (NCT00866281, NCT03591510). The latter is extremely important because in 2017, the FDA approved Midostaurin in combination with chemotherapy for newly diagnosed FLT3-mutated adult AML based on data of a multi-institutional, randomized phase 3 trial (RATIFY, NCT00651261). This trial showed that addition of Midostaurin to standard chemotherapy significantly prolonged overall and event-free survival among adult patients with AML and an FLT3 mutation (21). In children, Midostaurin is being evaluated as a single agent or in combination with chemotherapy in refractory/relapsed or newly diagnosed AML, respectively (NCT00866281, NCT03591510), and preliminary data indicated that single-agent Midostaurin, although adequately tolerated, has only limited clinical activity (22). However, since Midostaurin was the first drug approved in an AML genetic subgroup characterized by a specific gene mutation, this represents a starting point for novel therapy employing additional targeted agents, including second- and third-generation FLT3 inhibitors in ongoing clinical trials. Indeed, newer generation FLT3 inhibitors have a great specificity against FLT3, and consequently, they are more potent in inhibiting FLT3 with hopefully fewer off-target side effects. Among such compounds, Quizartinib, evaluated in relapsed childhood AML in combination with salvage chemotherapy, demonstrated a favorable toxicity profile and an encouraging response, consisting in complete FLT3 inhibition in all patients, and 4/17 and 10/17 complete remissions or stable disease, respectively (23). Currently, Quizartinib is being evaluated in a phase 1/2 study both in combination with re-induction chemotherapy and as a single-agent maintenance therapy in relapsed/refractory pediatric AML with FLT3-ITD mutations (NCT03793478). Crenolanib, a tyrosine kinase inhibitor developed as a selective and potent PDGFRα/β inhibitor, has also high affinity for FLT3, including both FLT3-ITD and FLT3-TKD mutations (24), and a phase 1 pilot study is currently assessing its toxicity profile in combination with Sorafenib in relapsed or refractory pediatric AML with mutated FLT3 (NCT02270788). Gilteritinib is a potent and selective FLT3 inhibitor with activity against both FLT3-ITD and FLT3-TKD mutations that demonstrated clinical efficacy in subjects with both wild-type or mutated FLT3 in phase 1/2 clinical trials enrolling adult patients with relapsed or refractory AML (25). In 2018, the FDA approved Gilteritinib as monotherapy to treat adult patients with relapsed/refractory AML and FLT3 mutations based on interim analysis of the ADMIRAL phase 3 trial (NCT02421939), which proved the superiority of Gilteritinib as compared to salvage chemotherapy in adults with relapsed and/or refractory FLT3 mutated AML. Indeed, the initial results of this trial reported a median OS significantly longer in the Gilteritinib arm than in the salvage chemotherapy arm (9.3 vs. 5.6 months), with 21% and 11% of complete remissions (CR) achieved in the two arms, respectively (26). Noteworthy, Gilteritinib is the first FLT3 inhibitor to be approved as monotherapy for AML patients. In 2016, the pediatric development program for Gilteritinib started, and in 2018, EMA approved several modifications to the pediatric investigation plan that now include two clinical studies evaluating Gilteritinib used in sequential combination with chemotherapy in pediatric patients from 6 months to <18 years of age with FLT3-ITD positive relapse/refractory AML (2215-CL-0603) or newly diagnosed AML (2215-CL-0604). Collectively, these trials provided important data regarding the efficacy of FLT3 inhibitors for AML treatment, and their application, particularly in combination with traditional chemotherapeutic agents as well as novel agents, would represent an important shift in the outcome of pediatric AML patients.

An additional gene frequently mutated in pediatric AML is *KIT* (7), a proto-oncogene that encodes a transmembrane glycoprotein type III receptor tyrosine kinase (RTK). The stem cell factor (SCF) promotes KIT dimerization and auto-phosphorylation that in turn lead to activation of complex downstream signaling pathways, including Ras/Erk, PI3K/Akt/mTOR, PLC-γ, Src kinase, and JAK/STAT signaling pathways, all essential to proliferation, differentiation, and survival of hematopoietic stem cells (27). In pediatric AML patients, *KIT* mutations occur in the extracellular portion of the receptor (exon 8), in the transmembrane domain (exons 10), in the juxtamembrane domain (exon 11), and in the activation loop of the tyrosine kinase domain (exon 17). These mutations affect RTK activity, due to ligand-independent activation of KIT, and tyrosine kinase inhibitors with activity against mutated KIT may represent effective therapeutic approaches. KIT mutations frequently associate with specific AML subtype, including core binding factor (CBF) AML. CBF-AML is characterized by the presence of aberrancies at CBF genes, and comprises t(8;21) and inv (16)/t(16;16), resulting in the *RUNX1-RUNX1T1* and *CBFB-MYH11* gene fusions, respectively. Both alterations affect CBF transcriptional complex, that is involved in the regulation of normal hematopoiesis, thus inducing leukemic transformation by blocking differentiation and promoting the self-renewal of stem cells and early progenitors (28). Collectively, CBF AML accounts about 20% of pediatric AML and is considered favorable (10). Accordingly, these patients receive a regimen of treatment based on four courses of chemotherapy (usually at lower dosages compared to other risk groups) not comprising HSCT (29). However, in some recent studies, the subgroup of t(8;21) AML patients showed a cumulative incidence of relapse of ~30%, similarly to high-risk patients (29–31). Although most of these patients have been subsequently rescued by an allograft, this resulting in an 8-year OS approaching 83%, the event free survival (EFS) remains unsatisfactory (29). These observation prompted to better investigate the impact of *KIT* mutation on prognosis of t(8;21) AML. Indeed, according to the multistep model of leukemogenesis, RUNX1-RUNX1T1 alone is not sufficient for leukemogenesis and requires co-operation with additional genetic hits, such as *KIT* mutations (32). In an interesting retrospective analysis of children with CBF-AML, Manara et al. found several differences between t(8;21) and inv(16)/t(16;16) AML, with a higher occurrence of *KIT* mutation in *RUNX1-RUNX1T1*- compared to *CBFB-MYH11*-rearranged patients (33). More importantly, t(8;21) AML with *KIT* mutations had a significantly worse prognosis than patients harboring only the translocation, suggesting that *KIT* mutations might contribute to the outcome and might be considered for risk stratification and therapeutically targetable markers in this subgroup of CBF-AML patients (33). Given the high frequency of *KIT* mutations and consequent elevated expression of this gene in AML with t(8;21), the addition to chemotherapy of the multikinase inhibitor Dasatinib has been evaluated in adult patients, and the results showed a favorable outcome (34). A recent phase 1 study is evaluating the clinical efficacy and tolerability of combination therapy of Dasatinib with multi-agent chemotherapy in relapsed child and adult AML patients with t(8;21) translocation and *KIT*^D816^ mutation (NCT03560908) (Table 1).

## Targeting Deregulated Signaling Pathways

In AML, uncontrolled proliferation and increased survival of leukemic cells can be sustained by deregulation of signal transduction pathways, whose components represent potential actionable targets.

Signaling regulated by Ras proteins are among the best characterized but most intricate signal transduction pathways in cell biology. Indeed, there are three members belonging to the Ras family (HRAS, KRAS, and NRAS), all found to be activated by mutations in human tumors, that play essential roles in controlling cellular functions involved in tumorigenesis including cell growth, division, differentiation, cell cycle regulation, cell migration, and angiogenesis (35). In addition, Ras proteins operate through two distinct pathways, the mitogen-activated protein kinases (MAPK) and phosphoinositide-3 kinase (PI3K) pathways (35). Collectively, mutations in both *NRAS* and *KRAS* genes account for more than 30% of pediatric AML patients, with a prominent age-related profile, and further RAS-related mutations, that affect RAS downstream components, may occur (7). On the other hand, PI3K/Akt/mTOR pathway deregulation occurs in a large proportion of AML patients. Its constitutive activation results from a variety of mechanisms besides Ras activating-mutations, including activating mutation in RTK (e.g., FLT3 and KIT mutations), mutations and/or over-expression in key signaling components (PI3K subunits, Akt, mTOR), alterations in the activity of the negative regulators PTEN and SHIP phosphatases, and deregulation in molecules that interact with this pathway (36). A plethora of compounds targeting Ras/MAPK and PI3K/Akt/mTOR signaling components were developed and tested in adult AML patients (37, 38); however, at present, only few of those inhibitors are investigated in pediatric AML patients (39). Since this review focuses on novel and promising targeted therapies potentially available for study in pediatric AML in the near future, an exhaustive review of all the Ras/MAPK and PI3K/Akt/mTOR signaling inhibitors is beyond our scope, and only few examples will be reported. Among the Ras/MAPK pathway inhibitors, Trametinib, a highly specific and potent MEK1/2 inhibitor, is currently investigated in children with Juvenile myelomonocytic leukemia (JMML) in a phase 2 trial (NCT03190915). Various clinical trials investigated FLT3 and KIT inhibitors, as above discussed, which in turn may down-modulate PI3K/Akt/mTOR signaling, and showed encouraging results. The PI3K/Akt/mTOR pathway inhibition can be achieved by targeting the key signaling components. In pediatric AML, both Akt (MK2206) and mTOR (RAD001) inhibitors were investigated in phase 1 clinical trials (NCT01231919 and NCT00081874, respectively). However, PI3K/Akt/mTOR inhibitors may induce significant toxicities, particularly in association with chemotherapy (39), without objective responses (40), thus limiting the clinical development of therapeutic approaches based on their application.

An interesting pathway deregulated in AML and recently investigated in adult patients is the Hedgehog (Hh) pathway, an evolutionary conserved process that regulates embryonic development and organ morphogenesis (41). Hh pathway has also been implicated in hematopoiesis, although its requirements depend on developmental stage (primitive or definitive hematopoiesis), cell maturation stage, and cell physiologic state, and a wide range of *in vitro* and *in vivo* studies demonstrated that targeting specific pathway components severely impairs proper hematopoiesis (42). Classical Hh pathway can be activated by one of three different ligands (Sonic Hedgehog, SHH; Indian Hedgehog, IHH; Desert Hedgehog, DHH) that bind the transmembrane receptor Patched (PTCH) that functions as a negative regulator of the pathway through inhibition of Smoothened (Smo). As a result of ligand binding, Smo is activated and induces a signaling cascade that culminate in the activation and nucleus translocation of GLI transcription factors that in turn regulate target gene expression (41). Due to its physiological role, it is not surprising that aberrant activation of Hh pathway is commonly observed in human cancers, including myeloid malignancies. Currently, the Smo inhibitors Sonidegib, Vismodegib, and Glasdegib are evaluated with multiple intervention approaches in phase 1, 2, and 3 clinical trials, enrolling adult patients with myeloid malignancies at different stages, including AML (43). In a phase 1 study, Glasdegib, administered as a single agent, demonstrated a biological activity in 16/28 adult AML patients by inducing one CR, four partial remissions, four minor responses, and seven stable diseases (44). A more recent randomized phase 2 clinical trial evaluated low-dose cytarabine plus/minus Glasdegib in newly diagnosed AML or high-risk myelodysplastic syndrome adult patients. Addition of Glasdegib increased median OS from 4.9 to 8.8 months, with complete remission rates of 17% for the Glasdegib arm vs. 2% for the standard arm, and a general favorable benefit–risk profile (45). Based on these results, in 2018, FDA approved Glasdegib in combination with low-dose cytarabine, for newly diagnosed AML patients who are 75 years old or older or who have comorbidities that preclude intensive induction chemotherapy. Several Smo inhibitors, including Sonidegib and Vismodegib, have been evaluated in pediatric patients affected by medulloblastoma (MB), the most common malignant brain tumor in children, demonstrating efficient HH pathway inhibition and anti-tumor activity (46). Although no evidence has still been reported in childhood AML, both results obtained in adult AML and pediatric MB patients prompted an extensive investigation of Hh inhibitors in the pediatric setting (47). With regard to Smo inhibitors, it should be taken into account the induction of permanent defects in bone growth, a toxicity profile that has been observed in both preclinical models and patients enrolled in clinical trials (46, 48). As a result, in 2016, the European Medicines Agency (EMA) modified the pediatric investigation plan of Glasdegib in pediatric patients with MB, with a waiver applied to the pediatric population from birth to <4 months of age. However, this drug-related toxicity was observed only with several compounds, including Glasdegib and Sonidegib (46, 48), thus supporting further investigation of additional Smo inhibitors especially in combinatorial drug regimens.

## Targeting Fusion Proteins

One of the most recurrent translocation in pediatric AML is *t*_(15, 17)_, which results in the fusion transcript *PML-RARA* and identifies a specific AML subtype, acute promyelocytic leukemia (APL), accounting for ~12% of pediatric AML (10). Among pediatric leukemias, APL is the first case where an effective targeted therapy was used, consisting of all-trans retinoic acid (ATRA) and arsenic trioxide (ATO), and this successful treatment radically changed APL from a detrimental to a curable malignancy, with complete remission rate over 90% (49, 50). These successful results constantly encourage the efforts in identifying effective targeted therapies against leukemia driver mutations.

The most common genetic events occurring in pediatric AML are the rearrangements of the *MLL* gene, accounting for 18% of patients (10), and in infants, the frequency is much higher, reaching 50% in several studies (3). MLL is a nuclear protein critical for hematopoietic development that normally regulates gene expression by catalyzing methylation of lysine 4 on histone 3 (H3K4). *MLL* rearranges with more than 80 different partner genes, and the resulting fusion proteins deregulate expression of MLL target genes. The most frequently overexpressed genes in *MLL*-rearranged leukemias are *HOX* cluster genes and the HOX cofactor *MEIS1*, which are normally expressed at highest levels only in the stem cells and early lineage progenitor cells, whereas they are down-regulated during differentiation (51). Although with several differences depending on fusion partner, the overall prognosis of *MLL*-rearranged AML is unfavorable (51). Great advancements in our knowledge of relevant mechanisms that determine the oncogenicity of MLL fusions have been made in recent years. The histone methyltransferase disrupter of telomeric silencing 1-like (DOT1L) is a methyltransferase that catalyzes methylation of lysine 79 on histone H3 (H3K79) and this epigenetic mark associates with active gene transcription (52). DOT1L acts in a multiprotein complex that also includes partners of MLL in the formation of MLL-fusion proteins (53). Therefore, in *MLL*-rearranged leukemias, DOT1L is recruited on MLL target sites, and its mislocated enzymatic activity promotes the leukemic gene expression program (54–56). It is not surprising that considerable efforts are being made to identify effective inhibitors to target DOT1L. Impressive results have been obtained in preclinical studies investigating pharmacologic inhibition of DOT1L (57), and the DOT1L inhibitor Pinometostat entered a phase 1 clinical trial to treat pediatric patients with relapsed/refractory leukemias bearing a rearrangement of the *MLL* gene (NCT02141828) (Table 1). However, despite Pinometostat biological activity and acceptable safety profile, no objective responses were observed (58). Currently, a phase 1/2 clinical trial evaluating Pinometostat in combination with standard chemotherapy to treat both child and adult patients with newly diagnosed *MLL*-rearranged leukemia is ongoing (NCT03724084) (Table 1).

An attractive therapeutic target for MLL-rearranged leukemias is represented by the menin–MLL interaction. Menin is a tumor suppressor protein that interacts with both wild type and rearranged MLL proteins and is required for the proper recruitment of MLL to the target genes. Because MLL-fusion proteins are difficult to target directly, pharmacological inhibition of the menin–MLL interaction is a promising therapeutic approach, since the leukemogenic activity of MLL fusion proteins is dependent on this interaction. In *in vivo* preclinical studies, the first orally bioavailable small-molecule inhibitors of the menin–MLL interaction, MI-463 and MI-503, resulted in growth inhibition and survival benefit in mouse models of MLL leukemia (59). In addition, further preclinical studies demonstrated that combining DOT1L and menin inhibition enhances the treatment efficacy in *MLL*-rearranged leukemia models (60). Interestingly, the synergistic effect of DOT1L and menin inhibitors has also been observed in AML models harboring *NPM1* gene mutations (61). Currently, two phase 1/2 clinical trials are evaluating the menin inhibitors KO-539 (NCT04067336) and SNDX-5613 (NCT04065399) in adult patients with relapsed/refractory AML or *MLL*-rearranged/*NPM1*-mutated AML, respectively.

*CBFA2T3-GLIS2* is a recently identified fusion transcript resulting from a cryptic inversion of chromosome 16 and specific to pediatric AML (62, 63). This chimeric oncogene identifies a peculiar subgroup of extremely aggressive pediatric AML with an incidence ranging between 9 and 30% among the whole cytogenetically normal (CN) AML (64) or the non-Down syndrome acute megakaryoblastic leukemia (non-DS-AMKL) subgroups (62), respectively. CBFA2T3 is a CBFA2T-family member that belongs to the RUNX1T1 complex and acts as a transcriptional co-repressor via its association with DNA-binding transcription factors, other corepressors, and histone-modifying enzymes, including the chimeric protein RUNX1-RUNX1T1 resulting from t(8;21) (64). GLIS2 (GLI-similar 2) is a member of the Kruppel-like zinc finger transcription factor group, which is closely related to the GLI family proteins, the transcription factors activated by the Hedgehog signal transduction cascade that regulate cell proliferation and self-renewal (64). Based on the homology between GLIS2 and GLI proteins, our group explored the possibility to target CBFA2T3-GLIS2 employing GANT61, a GLI1, and GLI2 inhibitor. Remarkably, *in vitro* treatment with GANT61 induced apoptosis in *CBFA2T3-GLIS2-*positive AML cells and reduced the expression of GLIS2-specific signature genes (65). Even if preliminary, these results are encouraging and prompt to extend the investigation of GLI inhibitors as a promising strategy to treat CBFA2T3-GLIS2 AML. Due to the discovery that induction of polyploidization and differentiation mediated by Aurora kinase A (AURKA) provides a therapeutic strategy for AMKL (66), Thiollier et al. investigated the efficacy of the AURKA inhibitor Alisertib (MLN8237) in a xenograft model of human AMKL expressing the CBFA2T3-GLIS2 fusion, demonstrating that Alisertib efficiently inhibits leukemic blast proliferation and increases survival, because of induction of terminal differentiation and apoptosis (67). However, a phase 2 clinical trial documented a poor response rate in children and adolescents affected by solid tumors or leukemia and receiving Alisertib as a single agent (68).

## Targeting Epigenetic Regulators

A common feature of AML is an altered epigenetic pattern resulting from both somatic mutations in epigenetic regulators or specific translocations that interfere with the normal epigenetic program (69). As a whole, mutations in epigenetic regulators are frequent in adult AML while they are uncommon in pediatric AML. For example, mutations in the NADP^+^-dependent isocitrate dehydrogenase genes 1 and 2 (*IDH1* and *IDH2*) were found in up to 33% adult AML and only in 3–4% of pediatric AML (70–72). Other epigenetic regulators mutated in pediatric AML, albeit rarely, are *TET2, DNMT3A*, and *ASXL1* (73). A relevant finding that emerged from these studies is the frequent co-occurrence of mutations in epigenetic regulators with other genetic anomalies in signaling transduction pathways and hematopoietic transcription factor genes, suggesting that mutations in epigenetic regulators cooperate in leukemogenesis. The last point also implies the possibility to combine treatments that target different class of mutations to eradicate the leukemic clone. In this regard, targeting the methyltransferase DOT1L is particularly intriguing for several reasons. Firstly, DOT1L is the only known methyltrasferase that catalyze mono-, di-, and tri-methylation of H3K79, and only recently a histone demethylase that can catalyze the removal of di- and tri-methyl groups from the H3K79 lysine residue has been identified (74); therefore, most likely DOT1L plays an essential role in AML cells. Next, DOT1L has diverse functions in mammalian cells, since methylation of H3K79 is primarily linked to active gene transcription and transcription elongation, but DOT1L also acts in DNA damage response and cell cycle regulation (53). Then, DOT1L associates with several complexes, including the elongation complexes EAP (75), SEC (76), and AEP (77), which, in turn, contain other proteins with oncogenic/leukemogenic activity. Finally, although the involvement of DOT1L in leukemia was initially linked to its mislocation due to MLL fusion proteins, some of the most recent works demonstrated a role of DOT1L also in non MLL-rearranged leukemias. For example, in preclinical studies, pharmacologic inhibition of DOT1L has been reported to impair proliferation, to induce cell differentiation, or to impact gene expression of primary AML cells harboring IDH1/2 mutations (78) as well as AML cell lines and a xenograft model with partial tandem duplication (PTD) of MLL (*MLL*-PTD) (79). DOT1L inhibition has been explored as a therapeutic target for the treatment of *DNMT3A* mutant AML, wherein reversed *DNMT3A*^mut^ induced gene activation and resulted in apoptosis and cell differentiation induction in both *in vitro* and *in vivo* AML models (80, 81). Finally, DOT1L inhibition has also been assessed in *NPM1* mutant AML and resulted synergistic with menin–MLL inhibitors in suppressing *HOX, MEIS1*, and *FLT3* gene expression and inducing AML cell differentiation (61). Overall, these studies demonstrated a remarkable role of DOT1L in AML cells irrespective of MLL fusion proteins, and based on these preclinical results, further investigation of combination treatments employing DOT1L inhibitors is warranted.

A further recently identified epigenetic target is the Bromodomain and Extra-Terminal Domain (BET) family of proteins, which includes BRD2, BRD3, BRD4, and BRDT proteins. This is the most prominent group of epigenetic reader proteins that regulates gene transcription by interacting with acetylated histones, thus facilitating transcriptional activation. Different small-molecule BRD inhibitors were developed and tested in cancers characterized by altered histone acetylation and aberrant gene transcription, including AML wherein epigenetic alterations are common (82). BET inhibitors demonstrated an anti-leukemic activity in preclinical models as both single agents or in combination with other drugs. In addition, their application is currently investigated in several clinical trials enrolling adult AML patients (82, 83), encouraging their application for pediatric AML treatment.

## Immunotherapy

A promising treatment to fight cancer is immunotherapy, an approach that exploits components of the immune system. There are several types of immunotherapy, including monoclonal antibodies, T-cell therapy, cancer vaccines, and other non-specific immunotherapies (84). All these therapeutic strategies have been employed to treat different types of cancer, including AML (85). In pediatric AML, the most relevant immunotherapy-based approach is represented by targeted therapy directed against surface antigens, in particular CD33 (sialic acid-binding immunoglobulin lectin, SIGLEC) and CD123 (IL3Rα), that are highly expressed, albeit not exclusively, on AML cells. A wide range of clinical trials were conducted to investigate the efficacy of gentuzumab ozogamycin (GO, Mylotarg) treatment in pediatric AML. GO is a monoclonal antibody to CD33 conjugated with the cytotoxic antibiotic calicheamicin that specifically induces cell death in CD33-expressing cells. GO originally received accelerated approval in 2000 as a stand-alone treatment for older patients with CD33-positive AML who had experienced a relapse, but it was withdrawn from the market after subsequent confirmatory trials failed to verify clinical benefit and demonstrated safety concerns, including a high number of early deaths. Nevertheless, in 2017, FDA approved GO for the treatment of adults with newly CD33-positive AML and patients aged 2 years and older with CD33-positive AML who have experienced a relapse or who are refractory (86). The newest FDA approval includes a lower dosing regimen, which induces less adverse events and is active for induction of remission, without curative intent (86). GO as monotherapy demonstrated a limited efficacy in children with relapsed/refractory AML (87) and did not delay the time to relapse when administered in postconsolidation therapy (88). However, GO was effective in reducing MRD levels in pediatric AML patients, when administered in combination with chemotherapy (89), and this is particularly beneficial in conditioning regimen prior to HSCT (90). The use of GO was also explored in consolidation targeted immunotherapy following HSCT (91). CD33-expressing AML cells may also be targeted by the unconjugated antibody Lintuzumab that was investigated in relapsed/refractory pediatric AML in phase 1 clinical trials (NCT00002890, NCT00672165). An exhaustive review describing all the other immunotherapies investigated in pediatric AML was recently published (92).

## Conclusions

**Figure 1:**
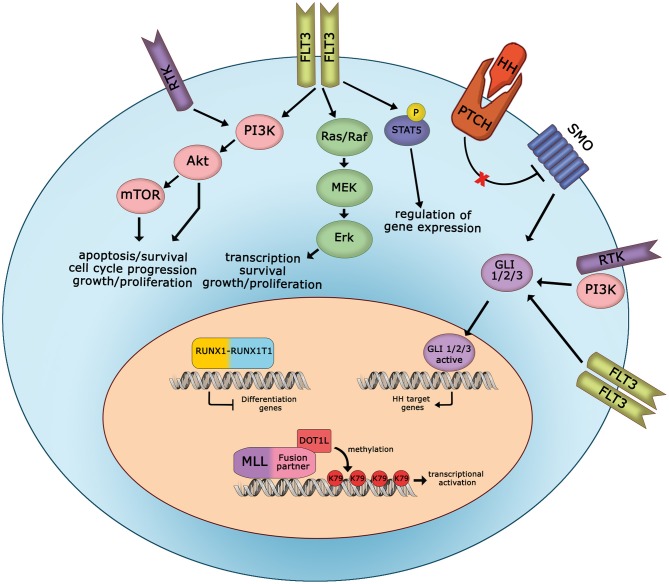
Cooperation between oncogenic signaling pathways in pediatric AML. Schematic representation of deregulated signaling and mutated proteins, involved in AML cell proliferation and survival, that can be targeted in pediatric patients. HH, hedgehog; RTK, receptor tyrosine kinase.

After decades of therapeutic advances for AML based almost exclusively on optimizing older drugs, since 2017, the field of novel therapies for AML has been rapidly developing. A major point to take into account is that lots of novel target inhibitors might be associated with each other or with conventional chemotherapy to increase treatment efficacy. Indeed, in AML, the genomic lesions often cooperate (Figure 1). For example, one of the most significantly up-regulated gene in *MLL*-rearranged leukemias is *FLT3*. Further, *NPM1-*mutated AML has aberrant *HOX* expression and frequently concomitant *FLT3* mutations. Also, the presence of fusion proteins interferes with normal cellular functions, and the efficacy of fusion protein targeting might be increased with additional drugs. In conclusion, such progresses in drug development, together with continuous efforts in our understanding of the molecular landscape of AML, provide great hope that more effective treatments may be offered to pediatric AML patients in the near future.

## Author Contributions

All authors contributed to the conception and editing of this review and approved the final manuscript. AL conducted literature review and wrote the manuscript in consultation with RM. AP critically revised the work. RM provided final approval of the version to publish.

### Conflict of Interest

The authors declare that the research was conducted in the absence of any commercial or financial relationships that could be construed as a potential conflict of interest.
